# Circulating MicroRNAs in non-small cell lung cancer: Clinical potential, analytic pitfalls, and barriers to implementation

**DOI:** 10.1016/j.jlb.2026.100497

**Published:** 2026-09-25

**Authors:** Iolanda Augustin, Adelina Birceanu, Laura Mazilu, Veronica Manolache, Ileana Constantinescu

**Affiliations:** a Faculty of Medicine, “Carol Davila” University of Medicine and Pharmacy, Bucharest, Romania; b Nord Hospital, Bucharest, Romania; c Pathoteam Diagnostic Center, Bucharest, Romania; d Ovidius University of Constanta, Constanta, Romania; e Ovidius Clinical Hospital, Constanta, Romania; f Department of Thoracic Surgery, Monza Hospital, Bucharest, Romania; g Center of Immunogenetics and Virology Fundeni, Fundeni Clinical Institute, Bucharest, Romania; h “Emil Palade” Center of Excellence for Young Researchers (EP-CEYR), Academy of Romanian Scientists, Bucharest, Romania; i Academy of Romanian Scientists, Bucharest, Romania

**Keywords:** Circulating microRNAs, Non-small cell lung cancer, Neoadjuvant chemoimmunotherapy, Predictive biomarkers, Liquid biopsy, Pathologic complete response

## Abstract

**Background:**

Neoadjuvant chemoimmunotherapy is standard care for resectable non-small cell lung cancer (NSCLC), yet 70% to 80% of patients do not achieve a pathologic complete response, and no validated biomarker identifies likely non-responders before or during treatment. Circulating microRNAs (miRNAs) have been proposed as non-invasive prognostic and predictive biomarkers, but their value in this setting remains largely undefined.

**Main body:**

Prognostic associations exist for miR-21, let-7, and miR-34a, with the miR-200 family reported inconsistently, and emerging predictive value for chemotherapy and checkpoint inhibitor response. A five-miRNA whole-blood signature, miRisk, stratified overall survival in two immunotherapy cohorts (n = 195) and outperformed PD-L1 TPS in both. In a chemoimmunotherapy control cohort (n = 139), the association with survival was not significant, a dissociation consistent with immunotherapy-specific rather than purely prognostic value. miR-34a, the miR-200/141 cluster, and miR-197 regulate the PD-1/PD-L1 axis directly in tissue and preclinical models, providing a mechanistic basis for immunotherapy-specific prediction. Whereas circulating tumor DNA captures tumor genotype and burden, miRNAs may additionally reflect the functional state of tumor and immune interactions, on which chemoimmunotherapy response depends. Demonstrating this requires paired tissue and plasma measurement, which is lacking. In the neoadjuvant setting, evidence is limited to one small proof-of-concept study, leaving pathologic response prediction open. Translation is constrained by pre-analytical heterogeneity, the absence of a consensus normalization strategy, and the near-complete absence of prospective validation.

**Conclusion:**

Circulating miRNAs complement ctDNA, but their clinical potential remains unrealized. Closing this gap will require mechanistically selected multi-miRNA panels, measured serially, integrated with ctDNA, and tested in adequately powered studies that standardize methodology from the outset.

## 1 Background

### 1.1 The unmet need of biomarker landscape in NSCLC

Lung cancer carries the heaviest burden of any malignancy worldwide. GLOBOCAN 2022 recorded approximately 2.48 million new cases and 1.82 million deaths, 12.4% of global cancer incidence and 18.7% of cancer mortality [1]. Non-small cell lung cancer (NSCLC) accounts for roughly 80% to 85% of these cases [2]. Despite two decades of therapeutic advances, five-year survival across all stages remains near 28%, reaching approximately 65% only when disease is localized at diagnosis [3].

The treatment landscape for resectable disease has shifted substantially. Platinum-based neoadjuvant or adjuvant chemotherapy long offered only a modest gain, on the order of 5% absolute improvement in five-year survival over surgery alone [4]. CheckMate-816 changed that: adding neoadjuvant nivolumab to platinum-doublet chemotherapy extended event-free survival from a median of 20.8 to 31.6 months (HR 0.63; 97.38% CI 0.43-0.91; P = 0.005) and raised the pathologic complete response (pCR) rate from 2.2% to 24.0% [5]. The final five-year analysis confirmed a significant overall survival benefit (HR 0.72; 95% CI 0.523-0.998; five-year OS 65.4% versus 55.0%), establishing neoadjuvant chemoimmunotherapy as a standard of care [6].

Several perioperative trials have since reinforced this. KEYNOTE-671 showed that perioperative pembrolizumab improved event-free survival versus chemotherapy plus placebo (HR 0.58; 95% CI 0.46-0.72), with pCR rates of 18.1% versus 4.0% [7]; a subsequent analysis confirmed an overall survival benefit (median OS not reached versus 52.4 months; HR 0.72; 95% CI 0.56-0.93) [8]. CheckMate-77T and AEGEAN, testing perioperative nivolumab and durvalumab, reported comparable pCR rates of 17% to 25% [9,10]. A meta-analysis of 43 studies and 5431 patients confirmed the pattern across the eight included randomized trials: pooled hazard ratios of 0.65 for overall survival and 0.59 for event-free survival, and a risk ratio of 5.52 for pCR [11].

Marinelli et al. (2025) showed that patients achieving pCR or major pathologic response (MPR, less than or equal to 10% residual viable tumor) have better event-free survival regardless of adjuvant immunotherapy [12]. Pathologic response therefore behaves as a reliable surrogate endpoint, which makes the clinical question concrete: who will achieve it? Roughly two-thirds of patients do not, and no validated biomarker reliably identifies likely non-responders before or during treatment.

### 1.2 Limitations of current biomarkers (PD-L1, TMB)

The two biomarkers most widely used to guide immunotherapy decisions in NSCLC, PD-L1 tumor proportion score and tumor mutational burden (TMB), carry well-documented shortcomings that are amplified in the neoadjuvant setting.

PD-L1 expression, measured by immunohistochemistry, accurately predicts immunotherapy outcomes in only about 30% of cases [13]. Expression is heterogeneous within tumors and across metastatic sites, so a single biopsy may misrepresent the broader immunological picture. Four antibody clones are in clinical use (22C3, 28-8, SP142, SP263), they do not always agree, and cut-off values differ between trials and agents [14].

The neoadjuvant data show the problem concretely. In the Sorin et al. (2024) meta-analysis, patients with PD-L1 greater than or equal to 50% derived the largest benefit (EFS HR 0.40; 95% CI 0.28-0.56), yet patients with PD-L1 <1% also benefited substantially (EFS HR 0.74; 95% CI 0.62-0.89) [11]. Pathologic complete responses occur across every PD-L1 stratum in the major trials, and PD-L1 enriches for higher response rates without predicting pathologic response at the individual patient level [15].

TMB carries analogous problems: no consensus sequencing panel, no standardized threshold, and a clinical significance that shifts with treatment context. Neither biomarker adequately predicts pathologic response to neoadjuvant chemoimmunotherapy, leaving a gap where reliable prediction would matter most [[14], [15], [16]].

### 1.3 The rationale for circulating miRNAs as liquid biopsy biomarkers

These limitations have driven interest in liquid biopsy: blood-based assays that can be repeated without invasive procedures. Among the circulating analytes under investigation (ctDNA, circulating tumor cells, exosomal cargo), microRNAs (miRNAs) are attractive for several practical reasons [17,18].

MiRNAs are short non-coding RNAs (19 to 25 nucleotides) that regulate gene expression post-transcriptionally. In circulation they travel inside extracellular vesicles, bound to AGO2 protein complexes, or associated with high-density lipoproteins; these carrier forms confer strong resistance to RNase degradation, extreme pH, and freeze-thaw cycles. They can be quantified by qRT-PCR, which is cheap, fast, and widely available [19,20].

The stronger argument is biological. Whereas ctDNA captures tumor genotype and mutational burden, miRNAs may reflect the functional regulatory state of both tumor and host cells [13,21]. In a treatment setting where outcome depends as much on the quality of the immune response as on cytotoxic killing, that functional layer may be exactly what current predictive models lack.

The literature was identified through PubMed/MEDLINE and Scopus searches for articles published between 2004 and 2026, combining the terms “circulating microRNA,” “miRNA,” “non-small cell lung cancer,” “liquid biopsy,” “prognostic biomarker,” “predictive biomarker,” “immune checkpoint inhibitor,” and “neoadjuvant chemoimmunotherapy,” with reference lists of relevant articles screened for additional sources. Priority was given to peer-reviewed primary studies, meta-analyses, and high-impact reviews. Consistent with the narrative scope of this review, study selection was not systematic.

Evidence on circulating miRNAs in the neoadjuvant setting remains minimal. No prospective study has yet validated a circulating miRNA panel for pCR or MPR under neoadjuvant chemoimmunotherapy in an adequately powered, externally validated design; none has used serial measurement across the treatment window; and the predictive value of whole-plasma (non-exosomal) panels in this setting is untested. This review evaluates circulating miRNAs as prognostic and predictive biomarkers in NSCLC, examines the methodological obstacles that have slowed clinical translation, and defines this gap as the rationale for prospective work.

## 2 Biology of circulating miRNAs: from biogenesis to biofluid stability

### 2.1 miRNA biogenesis

RNA polymerase II transcribes miRNA genes into primary transcripts (pri-miRNAs), which the nuclear Microprocessor complex (Drosha and DGCR8) cleaves into precursor miRNAs (pre-miRNAs) of approximately 60 to 70 nucleotides. After Exportin-5-mediated transport to the cytoplasm, Dicer and its cofactor TRBP process these into a mature duplex of roughly 22 nucleotides, and the guide strand is loaded into AGO2 to form the RNA-induced silencing complex (RISC), which directs translational repression or degradation of target mRNAs [22].

Not all miRNAs depend on the full cascade: mirtrons are spliced directly from short introns and enter at the Exportin-5 step, bypassing Drosha [22,23], and miR-451, essential for erythropoiesis, bypasses Dicer entirely and is cleaved by AGO2 [24]. These routes operate independently of individual processing components.

That independence is not trivial in NSCLC, where the canonical machinery is itself frequently disrupted. Reduced expression of both Dicer and Drosha has been documented in lung cancer, lower Dicer levels correlating with worse prognosis [24]. Epigenetic silencing of miRNA loci and dysregulation of Exportin-5 compound the effect, producing the global miRNA downregulation seen across many solid tumors, and this disruption of miRNA homeostasis provides a molecular basis for the altered circulating profiles detected in NSCLC patients [24,25].

### 2.2 Mechanisms of miRNA release into circulation

Circulating miRNAs reach the bloodstream through three principal carrier forms, and the relative contribution of each has consequences for biomarker design.

The quantitatively dominant form is also the simplest: miRNAs bound to AGO2 protein complexes, which account for approximately 90% of the circulating pool [26,27]. Arroyo et al. (2011) showed by differential centrifugation and size-exclusion chromatography that circulating miRNAs in human plasma partition predominantly with soluble protein fractions rather than with vesicles, and that protease digestion of plasma proteins collapsed miRNA stability, establishing AGO2 binding as what shields these molecules from blood RNases. How AGO2-miRNA complexes reach the circulation remains incompletely understood. The likeliest explanation is passive release: cells die, their contents spill out, and AGO2 is stable enough to persist extracellularly. Active secretion has not been demonstrated, though it cannot be excluded. The remaining ∼10% is encapsulated within extracellular vesicles (EVs), a minority fraction that has attracted considerable attention [26].

Villarroya-Beltri et al. first showed that exosomes transfer functional miRNAs between cells, and subsequent work revealed that sorting into EVs is actively regulated, through the ESCRT pathway, hnRNP A2/B1, ceramide-dependent mechanisms, and sequence motifs within the miRNAs themselves [28]. Because this sorting responds to oncogenic signaling, the EV-encapsulated fraction may carry a more tumor-specific signal than the passively released AGO2-bound pool [29].

A third, minor carrier form involves HDL-associated miRNAs, which can be delivered to recipient cells with functional gene-regulatory consequences [20].

A recent advance illustrates this directly. Ko et al. identified CD147-positive EVs as a miRNA-rich subpopulation that is overwhelmingly cancer-cell-derived: in xenograft models, 75% to 81% originated from tumor cells, against a minority for classical tetraspanin-positive EVs, and they contained 8- to 26-fold more miRNA, the enrichment mediated through a CD147-hnRNP A2/B1 interaction. miRNAs isolated by CD147 immunocapture from patient plasma mirrored the tumor tissue signature more closely than those obtained by conventional methods. If validated in larger cohorts, CD147-based isolation could address one of the more persistent limitations in this field: dilution of tumor-derived signal by miRNAs of non-malignant origin [29].

### 2.3 Stability of circulating miRNAs and implications for clinical application

For any circulating analyte to serve as a clinical biomarker, it must survive the interval between blood draw and laboratory measurement. Circulating miRNAs pass this test. Mitchell et al. showed that endogenous plasma miRNAs withstand RNase A incubation, room temperature storage for 24 h, up to ten freeze-thaw cycles, and pH extremes from 1 to 13 [19]. Synthetic miRNAs spiked unprotected into the same samples and degraded rapidly, which confirms that stability comes from the carrier, predominantly the AGO2 complex, rather than from anything intrinsic to the RNA molecule [26]. EV encapsulation and HDL association protect their fractions in the same way [20].

This has direct practical consequences. miRNAs can be quantified from standard serum or plasma processed through routine clinical workflows, without specialized preservation reagents, and archived biobank specimens remain informative, which opens the door to the large-scale retrospective validation studies the field still lacks [19]. The molecules surviving intact is not the whole story, however: as detailed below, hemolysis, platelet contamination, specimen type, and processing delays can all shift measured miRNA profiles considerably [19,25].

## 3 Methodological considerations in circulating miRNA quantification

Circulating miRNAs have been associated with NSCLC outcomes across hundreds of studies, yet none of these findings has translated into a clinical assay. The conflicting results that run through the literature are not purely biological in origin. A substantial part of the variability is technical: pre-analytical handling differs from one cohort to the next, analytical platforms are not interchangeable, and normalization strategies remain non-standardized. Reading the existing evidence accurately, and designing studies that could reach clinical-grade reliability, both depend on taking these variables seriously.

### 3.1 Pre-analytical variables

The largest source of variability in circulating miRNA measurement is also the one least likely to be reported: pre-analytical sample handling. An estimated 60% to 70% of laboratory errors in molecular diagnostics originate in this phase, and the sensitivity of qRT-PCR means that even minor differences in how a sample is collected or processed can alter the final result [30,31].

Specimen type matters from the outset. Serum and plasma yield quantitatively distinct miRNA profiles because the coagulation process releases miRNAs from platelets and other blood cells, producing systematically higher concentrations of certain miRNAs in serum. EDTA-anticoagulated plasma is generally preferred because it blocks coagulation without interfering with downstream enzymatic reactions. Heparin, by contrast, is a potent inhibitor of reverse transcriptase and DNA polymerase, and at typical concentrations it makes heparinized samples unsuitable for standard miRNA qRT-PCR workflows [30,32].

Hemolysis is the pre-analytical confounder most often overlooked in this field. Red blood cells are rich in specific miRNAs (for example miR-451a, miR-16, miR-486-5p, and miR-92a), and even low-grade hemolysis that is visually imperceptible can shift measured expression of these species by several fold. Hemolysis distorts commonly used endogenous references such as miR-16 [30,33]. This creates a compounding problem: because miR-16 is frequently employed as a normalization reference, hemolysis can simultaneously inflate the denominator and confound the numerator of expression ratios [33,34].

Time from blood collection to centrifugation is another variable that needs tight control. A processing window of under 2 h is recommended to limit leukocyte and platelet lysis. Double centrifugation protocols, an initial low-speed step followed by high-speed centrifugation at greater than or equal to 10,000 × g, are advocated for platelet depletion, since residual platelets can release miRNAs during freeze-thaw cycles and contribute to spurious signals [33]. Long-term storage at −80°C is the standard. Repeated freeze-thaw cycles, while tolerated by the miRNA molecules themselves, can disrupt residual cellular elements in incompletely processed samples and release intracellular miRNAs into the supernatant [32].

### 3.2 Analytical platforms

The miRQC study by Mestdagh et al., which systematically compared 12 commercially available miRNA profiling platforms, remains the reference cross-platform performance evaluation and provides the framework for understanding the strengths and limitations of current technologies [35].

Quantitative reverse transcription PCR (qRT-PCR) is treated as the gold standard in the field, on the basis of its sensitivity, specificity, and dynamic range, and it is the platform of choice for candidate-miRNA validation and clinical-grade assays. Its limitation is throughput: qRT-PCR requires pre-selected targets and is poorly suited to unbiased discovery. Two principal chemistries are in use, stem-loop RT primers (TaqMan-based) and poly(A) tailing with universal priming (SYBR Green-based), which show differential performance for specific miRNAs [35].

Next-generation sequencing (NGS), in the form of small RNA-seq, provides the broadest profiling capability, allowing detection of novel miRNAs, isomiRs, and post-transcriptionally modified species. The trade-offs are higher cost, demanding bioinformatics, and amplification and ligation biases introduced during library preparation that can distort quantification of individual miRNAs [35,36].

The dependence on reference genes and standard curves in qRT-PCR has driven interest in droplet digital PCR (ddPCR), which achieves absolute quantification by partitioning reactions into thousands of nanoliter-scale droplets. ddPCR is well suited to low-abundance targets, though throughput and per-assay cost remain limiting [37,38]. Amplification bias itself is addressed differently by the NanoString nCounter system, which bypasses enzymatic amplification through direct digital detection via color-coded molecular barcodes. The trade-off here is lower sensitivity for low-abundance miRNAs and restriction to pre-defined target panels [35].

A finding that recurs across all platform comparison studies is that cross-platform concordance breaks down for low-abundance miRNAs. The miRQC study showed that while all platforms reliably detected highly abundant species, performance diverged for miRNAs at low copy numbers, which is precisely where most cancer-specific circulating miRNAs sit [35]. This discordance undermines cross-study comparability and the reproducibility of published miRNA signatures.

### 3.3 Normalization strategies

In tissue-based miRNA studies, reference genes like RNU48 and U6 snRNA provide stable normalization anchors. No equivalent consensus reference exists for circulating miRNAs, and this absence is the single greatest technical barrier to clinical translation [34,39].

Three normalization approaches are in use, each with real limitations. Exogenous spike-in controls are synthetic non-human miRNAs such as *C. elegans* cel-miR-39, cel-miR-54, or cel-miR-238, added at defined concentration before extraction. They correct for technical variability in extraction efficiency and reverse transcription but cannot account for biological variation in total miRNA input, sample quality, or hemolysis [39].

Endogenous reference miRNAs rely instead on species presumed to be stably expressed across conditions. miR-16-5p and miR-223 are among the most commonly used normalizers, yet both are susceptible to hemolysis-induced elevation (miR-16 in particular, as one of the most abundant erythrocyte miRNAs) and both can be modulated by cancer and inflammatory states. An unstable reference introduces systematic error that cannot be detected without independent hemolysis assessment, a procedure many published studies have omitted [34,40].

Global mean normalization uses the average expression of all detected miRNAs as the normalization factor. This works well for high-throughput platforms profiling hundreds of miRNAs simultaneously, where the global mean is relatively resistant to changes in individual species. It is inappropriate, however, for candidate-gene qRT-PCR studies measuring only a small number of miRNAs, where the global mean becomes sensitive to the very changes under investigation [39,40].

Faraldi et al. showed that different normalization strategies applied to the same dataset yield divergent miRNA expression profiles [40], and Lakkisto et al. catalogued common methodological errors in circulating miRNA biomarker development, concluding that many published signatures may reflect normalization artifacts rather than genuine biological differences [34]. The poor reproducibility of miRNA signatures across independent cohorts is consistent with that assessment.

### 3.4 Toward standardization: current recommendations

Recent multi-center studies and expert consensus statements have begun to set minimum methodological standards for circulating miRNA research (Table 1).

**Table 1:** Proposed methodological quality standards for circulating miRNA biomarker studies in NSCLC.

Phase	Parameter	Recommended standard	Rationale
Pre-analytical	Specimen type	EDTA-anticoagulated plasma	Heparin inhibits RT-qPCR; serum introduces platelet-derived miRNA artifacts during coagulation [30,32]
	Centrifugation protocol	Double centrifugation: 1500–2000 × g for 10-15 min, then ≥10,000 × g for 10 min	Platelet depletion; residual platelets release miRNAs during freeze-thaw cycles [33]
	Processing time	Blood draw to centrifugation < 2 h	Leukocyte and platelet lysis releases intracellular miRNAs into plasma [33]
	Hemolysis assessment	Mandatory. miR-451a/miR-23a-3p ratio OR spectrophotometric OD at 414 nm (absorbance > 0.2 = significant hemolysis)	Hemolysis alters up to 50% of circulating miRNAs; miR-16, a common normalizer, is among the most affected [30,33]
	Hemolyzed samples	Exclude from primary analysis; include in sensitivity analysis if retained	Uncontrolled hemolysis introduces systematic error undetectable without independent assessment [34]
	Storage	−80°C; minimize freeze-thaw cycles	miRNA molecules tolerate freeze-thaw, but residual cellular elements in poorly processed samples do not [32]
Analytical	Discovery phase	NGS/small RNA-seq or high-throughput microarray	Unbiased profiling for candidate identification; acknowledge amplification and ligation biases in library preparation [35,36]
	Validation phase	qRT-PCR (TaqMan or SYBR Green)	Gold standard sensitivity and specificity for candidate validation; cross-platform concordance poor for low-abundance miRNAs [35]
	Absolute quantification	ddPCR when feasible	Reference-gene-independent; suited for low-abundance targets; limited throughput [37]
	Reporting	Specify platform, chemistry, primer design, and limit of detection	miRQC study demonstrated performance divergence across platforms specifically for low-abundance species [35]
Normalization	Strategy	Combination: exogenous spike-in (cel-miR-39) + empirically validated endogenous reference(s)	Spike-ins correct extraction efficiency; endogenous references correct biological input variation; neither alone is sufficient [33,39]
	Endogenous reference selection	Validate stability within each study population; do not adopt uncritically from prior publications	miR-16, miR-223 are hemolysis-sensitive and disease-modulated; published “stable” references may not generalize [34,40]
	High-throughput studies	Global mean normalization acceptable	Resistant to individual miRNA fluctuations when profiling hundreds of species simultaneously [39]
	Candidate-gene studies	Global mean normalization inappropriate	Global mean becomes sensitive to the changes under investigation when measuring few miRNAs [39,40]
Study design	Sample size	Adequate events-per-variable ratio for panel size; power calculation reported	Multi-miRNA panels in cohorts < 100 routinely violate EPV thresholds, inflating apparent performance [34]
	Validation cohort	Independent, geographically and/or temporally distinct from discovery	Internal cross-validation alone is insufficient; many reported signatures fail external replication [34]
	Response criteria	RECIST 1.1 (advanced setting) or pCR/MPR (neoadjuvant setting)	Unstated or inconsistent response criteria prevent cross-study comparison [41]
	Confounders	Match or adjust for age, sex, smoking status, comorbidities; report hemolysis status	A substantial proportion of published circulating miRNA biomarkers are highly expressed in blood cells [42]; uncontrolled confounders inflate or mask tumor signals
	Statistical approach	Treatment-biomarker interaction analysis for predictive claims; distinguish predictive from prognostic	A miRNA associated with poor outcomes in treated patients may reflect aggressive biology (prognostic) rather than treatment-specific resistance (predictive) [43]
	Reporting standards	TRIPOD + AI (Collins et al., BMJ 2024) for prediction models; REMARK for prognostic biomarkers	Rarely followed in circulating miRNA literature; essential for reproducibility and regulatory credibility [44]
	Inter-laboratory reproducibility	Ring trials with shared reference samples across participating sites	Certified reference materials for miRNA quantification do not exist; ring trials provide quantitative estimates of inter-institutional variability [45]

For specimen collection and processing, EDTA-plasma is the recommended specimen type. Double centrifugation (1500 to 2000 × g for 10 to 15 min, then greater than or equal to 10,000 × g for 10 min) should be performed within 2 h of collection, to ensure platelet-depleted, cell-free plasma. Hemolysis assessment should be done routinely, using either the miR-451a/miR-23a-3p ratio or spectrophotometric oxy-hemoglobin absorbance at 414 nm (absorbance >0.2 indicating significant hemolysis). Hemolyzed samples should be excluded or flagged for sensitivity analysis [30,33].

For normalization, the most defensible current approach combines exogenous spike-in controls with empirically validated endogenous reference miRNAs [33,39]. The choice of endogenous references should be validated within each study population rather than carried over from prior publications [34]. The miR-Test experience illustrates both the feasibility and the demands of clinical-grade standardization. After large-scale validation of a 34-miRNA serum signature in over 1000 screening participants, Marzi et al. used the same sample set to systematically optimize every step of the analytical pipeline, from RNA isolation and reverse transcription efficiency to inter-plate calibration and quality control checkpoints. That work showed that reproducible, clinical-grade circulating miRNA measurement is achievable, but only through the kind of prospective methodological development that most biomarker studies have not undertaken [46,47].

To date, no circulating miRNA assay has achieved regulatory approval from any major authority (FDA, EMA) for any cancer indication [34,48]. This stands in contrast to ctDNA-based assays, several of which have received FDA clearance for specific indications in NSCLC. The gap reflects not only biological complexity but the field's failure to achieve the pre-analytical, analytical, and normalization standardization that regulatory bodies require [30,33].

## 4 Circulating miRNAs as prognostic biomarkers in NSCLC

The prognostic utility of circulating miRNAs in NSCLC has been investigated in hundreds of studies over two decades. The evidence base is large but uneven: methodological rigor varies, effect sizes are inconsistent, and reproducibility across cohorts is limited, much of which traces back to the pre-analytical and normalization problems described above. What follows is an appraisal of the circulating miRNAs with the most developed prognostic associations, organized by functional class and by the strength of the underlying evidence, with the focus kept on serum and plasma species rather than tissue-based ones, except where tissue data are needed to interpret the circulating findings (Table 2).

**Table 2:** Summary of miRNAs with prognostic and predictive evidence in NSCLC.

miRNA	Direction	Sample type	N	Key metric	95% CI/P value	Validation	Ref
**A. Prognostic associations**							
miR-21	↑	Tissue (meta)	25 studies	HR 2.153 (NSCLC-specific OS)	1.693-2.739	Pooled meta-analysis	[49]
miR-21	↑	Circulating (meta)	Multiple	HR ∼2.37 (OS)	1.73-3.24	Pooled meta-analysis	[50]
miR-25-3p	↑	Serum	128 + 128	Sensitivity 76.4%, specificity 84.6%	Independent in multivariate	No external validation	[51]
miR-210	↑	Serum	2763 (pooled)	Associated with poor PFS, OS, DFS	Significant	GEO + TCGA mining	[52]
miR-155	↑	Circulating (meta)	Multiple	HR 1.42 (recurrence)	P = 0.007	Meta-analysis; OS not significant	[53]
let-7 family	↓	Tissue	143	HR 2.17 (OS, multivariate)	P = 0.009	No external validation	[54]
let-7f	↓	Plasma vesicle	-	Shorter OS	-	No external validation	[55]
miR-200c/141	↑	Tissue	155	miR-200c: P = 0.0004; miR-141: P = 0.009; combined OR 2.787	P = 0.002 (multivariate)	Adenocarcinoma only	[56]
miR-141	↑	Serum	108 + 54	AUC 0.856 (diagnostic/prognostic)	-	Single-center	[57]
miR-34a	↓	Plasma	196	Low miR-34a: shorter DFS (P = 0.011), OS (P = 0.011)	Not independent in multivariate	Single-center	[58]
**B. Predictive associations: Platinum-based chemotherapy**							
Exosomal miR-425-3p	↑ (resistant)	Exosomal (serum)	170	Inferior response, shorter PFS	-	Mechanistic validation (AKT1/autophagy)	[59,60]
miR-200c	↓ (resistant)	Preclinical	-	Restoration re-sensitizes to platinum	-	Cell line only	[61]
miR-25	↑ (resistant)	-	-	Cisplatin resistance via BIM/BAX	-	Review-level evidence	[62]
**C. Predictive associations: Immune checkpoint inhibitors**							
miRisk score (5-miRNA)	High score = poor ICI outcome	Whole blood	334 (96 train/99 valid/139 control)	HR 2.40 (OS, validation cohort)	1.37-4.19, P < 0.01	3 cohorts; not significant in chemo-IO control (HR 1.36, P = 0.37)	[13]
miR-183/96/182, miR-767/105	Predictive in *anti*-PD-1 LUAD	Plasma	12	Hypothesis-generating	-	No validation	[41]
EV-miR-625-5p	-	Exosomal	-	Independent predictor of ICI response at PD-L1>50%	-	Preliminary	[63]

### 4.1 Oncogenic miRNAs (OncomiRs) with prognostic significance

#### 4.1.1 miR-21

miR-21 is the most thoroughly studied oncomiR in NSCLC and among the first characterized as a circulating prognostic biomarker. It targets several tumor suppressors, including PTEN, PDCD4, and TPM1, and the net effect is promotion of proliferation, inhibition of apoptosis, and facilitation of invasion [64].

Meta-analytic data support its prognostic significance. Zhu and Xu pooled 25 studies and reported that elevated miR-21 was associated with worse overall survival across cancer types (HR 1.903, 95% CI 1.713 to 2.113), with the NSCLC-specific subgroup showing a stronger effect for tissue-based overexpression (HR 2.153, 95% CI 1.693 to 2.739) [49]. The circulating data are less stable than the tissue-based data: a separate meta-analysis by Wang et al. reported pooled HRs for poor OS of approximately 2.37 (95% CI 1.73 to 3.24), a comparable point estimate but with a wider confidence interval and substantial inter-study heterogeneity [50]. Tian et al. similarly found that miR-21 overexpression predicted advanced clinicopathological features and unfavorable prognosis in NSCLC specifically [65].

The problem is specificity. miR-21 overexpression is nearly universal across solid tumors and is elevated in inflammatory, cardiovascular, and metabolic disease [64]. That lack of disease specificity makes it weak as a standalone circulating NSCLC biomarker. Its prognostic value is therefore most likely to be realized inside multi-miRNA panels, where it can contribute an oncogenic signal alongside more NSCLC-specific species.

#### 4.1.2 miR-25-3p and the mir-25/93/106b cluster

The miR-25/93/106b cluster, encoded within intron 13 of MCM7 on chromosome 7, is a paralog of the oncogenic miR-17-92 cluster. Transcription is driven by YAP/TAZ, and the cluster promotes proliferation by suppressing the cyclin-dependent kinase inhibitor p21, keeping p21 levels low in NSCLC tissue relative to normal lung [66].

Two studies have evaluated circulating miR-25 in NSCLC. Li et al. reported that serum miR-25 distinguished NSCLC from controls with 76.4% sensitivity and 84.6% specificity (n = 128 per group), with high expression predicting shorter OS and relapse-free survival on multivariate analysis [51]. Zhang et al. found the same pattern: plasma miR-25 correlated with aggressive clinical features and poor survival, and levels dropped after surgical resection, which fits a tumor-derived origin [67]. The cluster has also been implicated in cisplatin resistance through modulation of the BIM/BAX apoptotic pathway [62].

#### 4.1.3 miR-210 and miR-155

miR-210, a direct HIF-1α target, is the most reliably hypoxia-induced miRNA. Serum miR-210 correlates with NSCLC stage and lymph node metastasis, drops in cisplatin responders [68], and a pooled analysis of 2763 samples confirmed its association with poor PFS, OS, and DFS [52].

miR-155 is a more complicated case. Circulating levels are elevated in NSCLC with good diagnostic accuracy (AUC 0.87 [69]), and Yang et al. linked high serum miR-155 to recurrence (pooled HR 1.42, P = 0.007), though the association with OS did not reach significance [53]. Pooled prognostic data are nevertheless inconsistent, and that fits the biology: miR-155 plays opposing roles depending on cellular context. In tumor cells, it drives proliferation and chemoresistance through SOCS1/PTEN suppression. In the immune microenvironment, it suppresses PD-L1 on tumor-associated macrophages, supports CD8^+^ T cell effector function, and regulates dendritic cell maturation [70,71]. Whether circulating miR-155 predominantly reflects tumor-derived or immune-derived signal, a distinction current assays cannot make, likely determines the direction of its prognostic effect.

### 4.2 Tumor-suppressive miRNAs with prognostic value

#### 4.2.1 The let-7 family

The let-7 family was among the first miRNA families discovered and the first linked to cancer prognosis. Takamizawa et al., working with tissue from 143 surgically resected lung cancers, showed that reduced let-7 expression was associated with shortened postoperative survival (P = 0.0003), with multivariate analysis confirming an independent prognostic impact (HR 2.17, P = 0.009). This was the first miRNA-prognosis association reported in any human cancer [54].

The family suppresses tumor growth by targeting KRAS, MYC, and HMGA2. In the circulation, reduced plasma vesicle-associated let-7f correlated with shorter OS in NSCLC patients, an early signal that the tissue-based prognostic association carries over to circulating measurement [55]. More recently, let-7b-5p has been identified within exosome-derived signatures capable of distinguishing both LUAD and LUSC from healthy controls [72]. Reduced circulating let-7 has also been linked to chemoresistance in lung cancer, with levels tracking treatment in real time: they rise after effective therapy and fall again at relapse [73]. That behavior positions the let-7 family as a candidate both for baseline prognostication and for treatment monitoring.

#### 4.2.2 The miR-200 family and miR-141

The miR-200 family (miR-200a/b/c, miR-141, miR-429; encoded in two clusters on chromosomes 1 and 12) regulates EMT through direct targeting of ZEB1 and ZEB2. Given the central role of EMT in NSCLC metastasis and drug resistance, this family has attracted sustained prognostic interest, but the literature is genuinely contradictory.

In tissue, Tejero et al. found that high miR-200c and miR-141 expression predicted shorter OS in 155 resected NSCLC patients, but only in adenocarcinoma (miR-200c: P = 0.0004; miR-141: P = 0.009), not in squamous cell carcinoma. A combined risk score based on miR-141 and miR-200c expression was independently prognostic on multivariate analysis, both in the overall cohort (OR 2.787, P = 0.033) and, with a much larger effect, in adenocarcinoma specifically (OR 10.649, P = 0.002). This histology dependence matters and is easily missed [56]. In circulation, serum miR-141 has been reported as elevated in NSCLC with diagnostic accuracy (AUC 0.856) and as an independent prognostic factor for OS, again selectively in adenocarcinoma [57]. A broader review nevertheless documented that serum miR-200 levels are inconsistently reported across NSCLC studies, elevated in some cohorts and reduced in others, with poor tissue-to-biofluid expression concordance [74].

This apparent contradiction is reconcilable, and doing so matters because miR-200c and miR-141 are central to the mechanistic model developed in Section 5.2.3. Three factors are at work. First, the direction of effect is histology-dependent: the unfavorable association reported by Tejero et al. was confined to adenocarcinoma, with no effect in squamous cell carcinoma [56], so any circulating measurement that pools histologies will blur it. Second, the tissue and circulating compartments need not move together. High intratumoural miR-200, an epithelial-state marker, restrains epithelial-to-mesenchymal transition at the primary site, yet the family also supports mesenchymal-to-epithelial reversion and colonisation at metastatic sites, so elevated tumor or circulating miR-200 can accompany aggressive, metastasising disease rather than contradict it. Third, the circulating signal is compartment-shifted and normalization-sensitive, and the poor tissue-to-biofluid concordance noted above [74] means the plasma level cannot be read as a direct surrogate for the tumor level. The practical consequence is that the direction of effect for circulating miR-200c and miR-141 is not yet established, and their inclusion in a predictive panel is justified on mechanistic grounds, as regulators of ZEB1 and PD-L1 (Section 5.2.3), rather than on a settled circulating association. Resolving the direction will require histology-stratified measurement with defined normalization and paired tissue sampling.

Diagnostically, miR-141 contributed to a 4-miRNA serum panel that discriminated early-stage NSCLC from controls with high accuracy [75]. Whether that diagnostic signal translates into prognostic utility in established disease remains open.

#### 4.2.3 miR-34a

miR-34a is a direct transcriptional target of p53 and regulates apoptosis, cell cycle arrest, and senescence. In circulation, Zhao et al. evaluated plasma miR-34a in 196 NSCLC patients and found that low plasma miR-34a correlated with lymph node metastasis (P = 0.002) and with shorter DFS (P = 0.011) and OS (P = 0.011) on univariate analysis. Tissue miR-34a carried stronger prognostic associations (DFS: P = 0.002; OS: P < 0.001). The plasma association did not retain independent significance on multivariate analysis, most likely because its expression tracked lymph node status, which itself drives prognosis. The authors concluded that circulating miR-34a may serve as a less invasive surrogate for tissue-based prognostication, accepting some loss of discriminatory power [58].

### 4.3 Multi-miRNA prognostic panels and signatures

The field has moved away from single-miRNA assessment toward multi-miRNA panels, on the reasonable assumption that no single species captures the biological complexity of NSCLC. Abdipourbozorgbaghi et al. quantified miRNA expression in plasma from 122 participants (78 NSCLC, 44 controls), with validation in an independent publicly available dataset of more than 4000 NSCLC patients [41].

For LUAD, they identified a seven-miRNA diagnostic panel, and separately found miR-135b-5p, miR-196a-5p, and miR-31-5p to be independent prognostic biomarkers in multivariate Cox regression. For LUSC, a nine-miRNA panel was proposed, with both strands of miR-205 acting as independent prognostic markers [41]. The fact that the two panels discriminate between the histological subtypes carries a methodological lesson. LUAD and LUSC are biologically distinct diseases, and treating them as a single entity in biomarker development risks obscuring subtype-specific signals.

## 5 Circulating miRNAs as predictive biomarkers for treatment response

A prognostic biomarker reflects overall patient outcome irrespective of the treatment administered. A predictive biomarker identifies which patients are likely to benefit from a specific intervention. The distinction is more consequential than it looks. Many circulating miRNAs studied in NSCLC carry prognostic associations (Table 2), but whether any of them are truly predictive, able to discriminate responders from non-responders to a given therapy, is a far harder claim to support, and one that most published studies do not address with the methodology it requires.

### 5.1 Prediction of platinum-based chemotherapy response

Platinum-based doublet chemotherapy remains central to systemic treatment of NSCLC, both in advanced disease and as the cytotoxic backbone of neoadjuvant chemoimmunotherapy regimens. Identifying circulating miRNA markers of platinum sensitivity or resistance is therefore clinically relevant across multiple treatment contexts [76].

The strongest evidence to date comes from exosomal miRNA profiling. Yuwen et al. used HiSeq deep sequencing of circulating exosomal miRNAs from 170 serum samples and identified six differentially expressed species (miR-425-3p, miR-1273h, miR-4755-5p, miR-9-5p, miR-146a-5p, and miR-215-5p) between platinum-resistant and platinum-sensitive NSCLC patients. Of these, exosomal miR-425-3p was the most mechanistically developed: it regulates platinum response through AKT1-mediated autophagy modulation, and elevated circulating levels correlated with inferior treatment response and shorter progression-free survival [59]. A follow-up by the same group showed that exosomal miR-425-3p levels rise over the course of platinum-based treatment, and that exosomes from cisplatin-resistant cells could confer chemoresistance to sensitive cells in a miR-425-3p-dependent manner [60]. This is one of the few cases in the circulating miRNA biomarker literature where a mechanistic pathway has been traced from a clinical observation through to a functional in vitro model.

Beyond exosomal species, miR-25 expression has been correlated with cisplatin resistance, in line with the proliferative and anti-apoptotic functions of the miR-25/93/106b cluster [62]. The miR-200 family has also been implicated through the EMT and drug resistance axis: loss of miR-200c permits ZEB1-mediated EMT and concomitant chemoresistance, and restoration of miR-200c re-sensitizes resistant cells to platinum agents in preclinical models. Whether these associations reflect specific platinum resistance mechanisms or simply more aggressive tumor biology is not yet clear [61]. That ambiguity has practical consequences. A circulating miRNA that correlates with poor outcomes in platinum-treated patients may be marking aggressive disease, a prognostic effect, rather than platinum-specific resistance, a predictive one. Establishing genuine predictive utility requires either treatment-by-biomarker interaction analyses in randomized trials or, failing that, evidence that the miRNA association is absent in an untreated or alternatively treated control arm. Few published studies in this area meet that standard, and the field is also constrained by predominantly single-center, retrospective designs with modest sample sizes and no external validation cohorts [13]. Normalization practice across these studies is likewise heterogeneous, which further limits cross-study comparison [77].

### 5.2 Prediction of immune checkpoint inhibitor (ICI) response

#### 5.2.1 Blood-based miRNA signatures for ICI outcome

Among the potential applications of circulating miRNA biomarkers, prediction of immunotherapy response is the one with the most pressing clinical demand. PD-L1 tissue protein staining accurately predicts ICI response in only about 30% of cases [13], TMB remains inadequately standardized across assay platforms, and no blood-based biomarker has yet achieved regulatory validation for this purpose [78].

The best-developed blood-based miRNA signature in this space is the miRisk score, reported by Rajakumar et al. (2022). This five-miRNA whole blood signature was developed using small RNA sequencing in a training cohort (n = 96) and tested in an independent validation cohort (n = 99) and a chemoimmunotherapy control cohort (n = 139), totaling 334 stage IV NSCLC patients. In the independent validation cohort, high miRisk was associated with shorter overall survival (HR 2.40, 95% CI 1.37 to 4.19, P < 0.01). At the 6-month timepoint, the time-dependent AUC of miRisk was 0.87 (95% CI 0.78 to 0.95) in the training cohort and 0.71 (95% CI 0.58 to 0.82) in the validation cohort. Expressed as the difference in time-dependent AUC relative to PD-L1 TPS, miRisk improved discrimination by 0.18 (95% CI 0.01 to 0.35) in the validation cohort and by 0.36 (95% CI 0.22 to 0.50) in the training cohort, the larger training estimate reflecting in-sample optimism. Adding PD-L1 TPS to the miRisk model did not improve performance, which indicates that miRisk captures information not redundant with PD-L1 [13].

The behavior of miRisk across cohorts speaks directly to the predictive versus prognostic distinction. High miRisk was associated with shorter overall survival in the two immunotherapy cohorts but not in the control cohort of 139 patients treated with chemoimmunotherapy, where the association was not significant (HR 1.36, P = 0.37) and likewise did not reach significance after multivariable adjustment [13]. A signal present under immunotherapy and absent under chemoimmunotherapy is the pattern a treatment-specific marker would produce, rather than one of general prognosis. A subsequent report from the same group, restricted to patients with PD-L1 TPS of at least 50%, reproduced this dissociation in independent cohorts: miRisk stratified survival under immunotherapy monotherapy (training n = 68, HR 3.84, 95% CI 1.86 to 7.95; validation n = 56, HR 5.37, 95% CI 1.96 to 14.74) but not under chemoimmunotherapy (n = 31, HR 1.41, 95% CI 0.17 to 11.91) [79]. Two qualifications keep this short of proof. Neither study reports a formal biomarker-by-treatment interaction test, and the original authors state that the predictive versus prognostic nature of the score has not been definitively addressed, since a chemotherapy-only control was unobtainable and the available control cohort also received immunotherapy. The chemoimmunotherapy cohorts are moreover small and imprecisely estimated, so the absence of an association is weak evidence for the absence of an effect. The implication for the present review is nonetheless direct: the only cohorts in which miRisk has been tested under combined chemoimmunotherapy did not reproduce the immunotherapy-cohort signal, and whether any circulating miRNA signature retains predictive value once chemotherapy is added to immunotherapy must therefore be established de novo rather than inherited from immunotherapy-monotherapy data.

In a complementary analysis, Abdipourbozorgbaghi et al. identified the miR-183/96/182 and miR-767/105 clusters as showing predictive potential in *anti*-PD-1-treated LUAD patients. The finding came from a small ICI-treated subcohort (n = 12 LUAD patients) and should be read as hypothesis-generating, but it raises the possibility that miRNA-based prediction could be histology-specific, a consideration that has received little attention in the literature [41].

Dynamic changes in circulating miRNA profiles during ICI therapy add a further dimension. Serial measurement studies have shown that specific circulating miRNAs shift in correlation with radiologic response per RECIST criteria, which suggests that miRNA kinetics, not static baseline levels alone, may capture the evolving tumor and immune interaction during treatment [80].

#### 5.2.2 Exosomal miRNAs and ICI resistance

Extracellular vesicle-associated miRNAs have also been investigated as predictors of ICI resistance. Pre-treatment upregulation of specific exosomal miRNAs has been associated with immunotherapy resistance in advanced NSCLC, and EV-miR-625-5p has been identified as an independent predictor of ICI response in patients with PD-L1 expression above 50% in preliminary analyses [63,81]. The biological rationale is straightforward: exosomes mediate intercellular communication between tumor and immune cells, and their miRNA cargo can directly modulate immune cell function in the tumor microenvironment.

Del Re et al. reported that an increase in exosomal PD-L1 mRNA copies during *anti*-PD-1 treatment, reflecting dynamic immune activation, correlated with clinical response in melanoma and NSCLC patients [82]. Shimada et al., in parallel, found that higher baseline serum exosomal PD-L1 levels were associated with favorable response to *anti*-PD-1 therapy in NSCLC, in a subgroup of 17 treated patients, which fits the idea that tumors most dependent on the PD-1/PD-L1 checkpoint for immune evasion are also the most vulnerable to its blockade [83]. The point matters because exosomal PD-L1 is a functional, circulating form of the checkpoint ligand that may participate in systemic immune modulation beyond the local tumor microenvironment [84]. Conflicting data nevertheless persist, both on the concordance between exosomal PD-L1 and tissue PD-L1 expression and on the direction of the association with treatment response. Until these discrepancies are resolved, ideally through paired tissue and exosome analyses in prospective cohorts, exosomal PD-L1 measurements should be interpreted with caution [83,85]. These candidates sit within a broader landscape of proposed biomarkers of immunotherapy resistance, none of which has yet reached clinical validation [86].

#### 5.2.3 miRNAs targeting the PD-1/PD-l1 axis

Beyond statistical associations, several miRNAs have been shown to regulate the PD-1/PD-L1 checkpoint pathway directly, which gives mechanistic weight to their candidacy as immunotherapy predictors. miR-34a is a direct transcriptional target of p53. In a series of experiments using NSCLC cell lines, patient tumor samples, and a syngeneic mouse model, Cortez et al. established that p53 regulates PD-L1 via miR-34a, which binds the 3′-UTR of CD274 (PD-L1) mRNA (mechanism of action summarized in Fig. 1). Luciferase reporter assays showed that miR-34a reduced PD-L1 expression by roughly 50% (mean fold-change 0.50, SD 0.2, P < 0.001). Analysis of 181 NSCLC samples from The Cancer Genome Atlas revealed an inverse correlation between p53 and PD-L1 expression (r = −0.29, P < 0.001), and tumors harboring p53 mutations had lower miR-34a and higher PD-L1 levels than wild-type tumors (P = 0.03). When the authors delivered miR-34a mimics via liposomal nanoparticles (MRX34) in a p53-mutant/K-ras-driven syngeneic NSCLC model, tumor PD-L1 expression decreased, CD8^+^ T-cell infiltration rose from 22.5% to 30.1% (P = 0.016), and the proportion of exhausted PD-1+CD8^+^ T cells was halved (40.2% to 20.3%, P = 0.001). The effect was amplified when MRX34 was combined with radiotherapy, yielding CD8^+^ levels of 44.2%, above either treatment alone [87]. Together these findings position circulating miR-34a as a candidate non-invasive surrogate for the tumor's capacity for PD-L1-mediated immune evasion, particularly in the sizeable subset of NSCLC patients carrying p53 loss-of-function mutations.

**Fig. 1:**
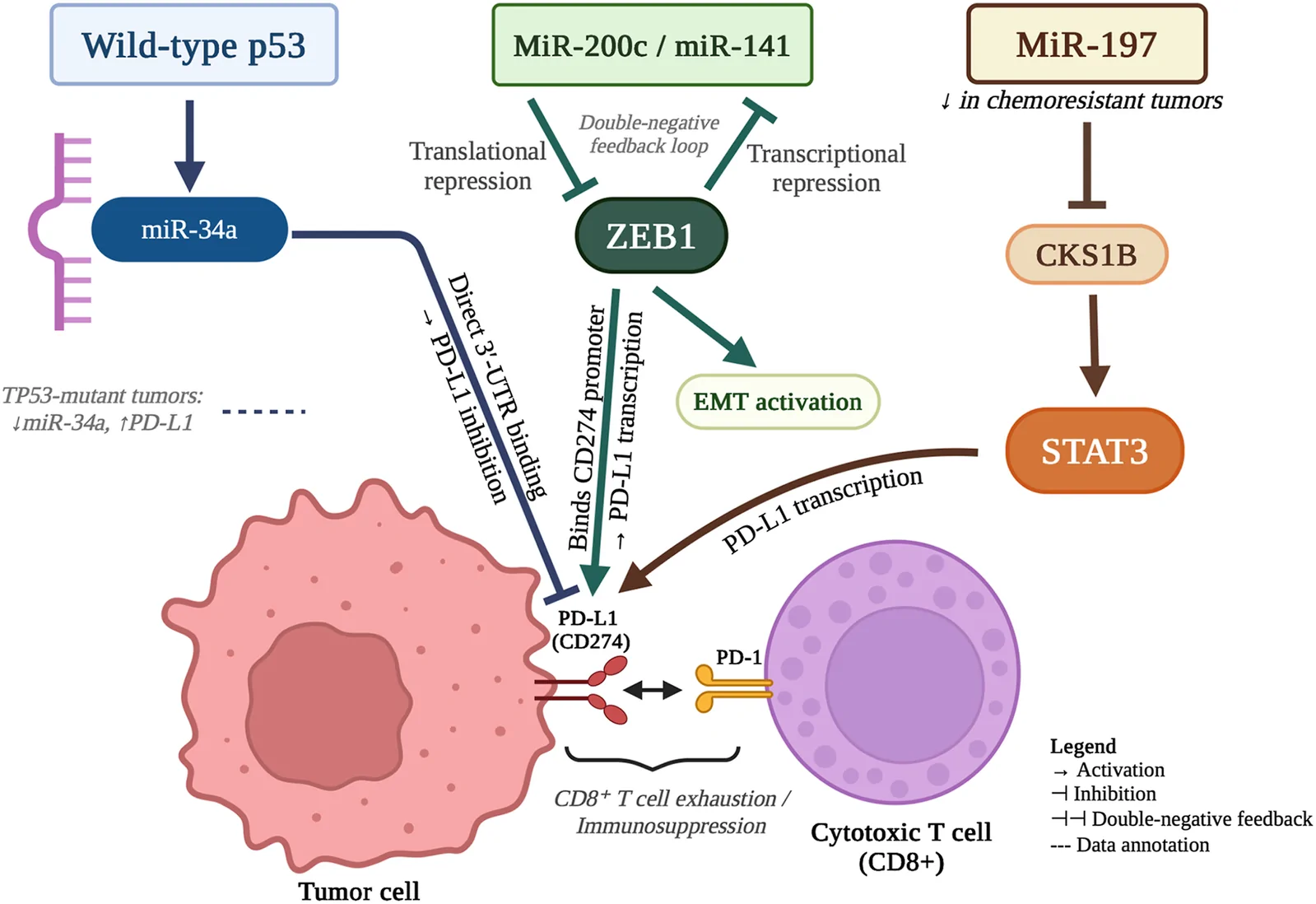
**Mechanistic convergence of three miRNA pathways on PD-L1 regulation in NSCLC.** Left (blue): p53 transcriptionally activates miR-34a, which directly binds the 3′-UTR of CD274 (PD-L1), reducing expression by approximately 50% (fold-change 0.50, P < 0.001). Analysis of 181 NSCLC samples from The Cancer Genome Atlas confirmed a significant inverse correlation between p53 and PD-L1 expression (r = −0.29, P < 0.001), with TP53-mutant tumors exhibiting lower miR-34a and higher PD-L1 (P = 0.03). In a syngeneic NSCLC model, liposomal miR-34a delivery (MRX34) increased CD8^+^ tumor-infiltrating lymphocytes from 22.5% to 30.1% (P = 0.016) and halved the proportion of exhausted PD-1^+^ CD8^+^ T cells (P = 0.001); the subsequent Phase 1 clinical trial was terminated due to serious immune-related toxicities (Cortez et al. [87]; Hong et al. [88]). Center (green): miR-200c/141 and ZEB1 form a double-negative feedback loop. ZEB1 transcriptionally represses miR-200, while miR-200 translationally represses ZEB1. Loss of miR-200 relieves ZEB1 suppression, simultaneously activating EMT and PD-L1 transcription via direct ZEB1 binding to the CD274 promoter (Chen et al. [89]; Guo et al. [90]). This circuit provides a dual readout of metastatic potential and immune evasion capacity from a single miRNA measurement. Right (orange): in chemoresistant NSCLC, miR-197 downregulation derepresses CKS1B, activating STAT3-mediated PD-L1 transcription. In 177 patients, miR-197 levels were inversely correlated with PD-L1 expression (P = 0.026) and overall survival (P = 0.015), linking platinum resistance and immune evasion through a shared signaling cascade (Fujita et al. [91]). All three pathways converge on PD-L1 upregulation and CD8^+^ T cell immunosuppression. Created in BioRender.

The therapeutic translation went badly: the subsequent Phase 1 clinical trial of MRX34 as a therapeutic agent was terminated after multiple immune-mediated serious adverse events, including four patient deaths attributed to distinct immune-related toxicities (hepatic failure, colitis/pneumonitis progressing to multi-organ failure, hypoxia/systemic inflammatory response syndrome, and cytokine release syndrome/respiratory failure) [88].

The miR-200/141 cluster regulates PD-L1 indirectly through its canonical EMT target ZEB1 (Fig. 1). ZEB1 functions both as an EMT inducer and as a transcriptional repressor of miR-200, and the loss of miR-200 lifts repression of PD-L1, with consequent CD8^+^ T-cell immunosuppression and metastasis. The study showed consistent correlations between EMT scores, miR-200 levels, and PD-L1 expression across multiple human lung cancer datasets. This ZEB1, miR-200, and PD-L1 regulatory circuit means that a single miRNA readout could in principle capture information about both metastatic potential (EMT status) and immune evasion capacity (PD-L1 regulation), a dual readout that no single protein biomarker currently offers, provided that circulating levels can be shown to track intratumoural levels [89]. Subsequent work by Guo et al. extended these findings, showing that ZEB1 binds directly to the CD274 promoter to enhance PD-L1 transcription and forms an immunosuppressive microenvironment around invading cancer cells [90].

miR-197 operates through a different mechanism. Fujita et al. identified a miR-197/CKS1B/STAT3-mediated PD-L1 network in chemoresistant NSCLC: miR-197 downregulation in platinum-resistant tumors leads to CKS1B/STAT3 axis activation, which drives both tumor progression and PD-L1 upregulation (Fig. 1). In a validation cohort of 177 patients, miR-197 levels were inversely correlated with PD-L1 expression (P = 0.026) and with worse overall survival (P = 0.015). The finding matters because it links chemoresistance and immune evasion through a shared miRNA regulatory cascade, the kind of dual-pathway involvement that becomes directly relevant in the chemoimmunotherapy setting [91]. Table 3 summarizes the main miRs involved in PD-1/PD-L1 axis regulation and their mechanism of action.

**Table 3:** Summary of circulating miRNAs with mechanistic links to PD-1/PD-L1 axis regulation.

miRNA	Mechanism	Key data	N	P value	Ref
miR-34a	p53 → miR-34a ⊣ PD-L1 (3′-UTR direct binding)	Luciferase fold-change 0.50; TCGA: r = −0.29 (p53 vs PD-L1); mouse: CD8^+^ T cells 22.5% → 30.1%	181 (TCGA)	< 0.001	[87]
miR-200/141	miR-200 ⊣ ZEB1 → ZEB1 activates PD-L1 transcription	EMT scores correlate with PD-L1 across lung cancer datasets; ZEB1 binds CD274 promoter	Multiple datasets	-	[89,90]
miR-197	miR-197 ⊣ CKS1B → STAT3 → PD-L1	miR-197 inversely correlated with PD-L1; connects chemoresistance + immune evasion	177	P = 0.026 (PD-L1); P = 0.015 (OS)	[91]

One caveat applies to this entire mechanistic model and needs to be evidentiated. The evidence that these miRNAs regulate PD-L1, summarized in Fig. 1 and Tables 3 and is tissue-based, preclinical, or TCGA-derived: luciferase reporter assays, syngeneic mouse models, and p53-PD-L1 correlations in tumor tissue. None of it demonstrates that the circulating level of miR-34a, miR-200c, or miR-197 reflects the intratumoural level of the same miRNA, which is the assumption required to move from mechanism to a blood-based predictive test. The evidence assembled in this review in fact cautions against taking that step for granted: plasma miR-34a loses independent prognostic significance on multivariable analysis while tissue miR-34a retains it (Section 4.2.3), serum miR-200 shows poor tissue-to-biofluid concordance (Section 4.2.2), and a substantial fraction of circulating miRNA biomarkers are highly expressed in blood cells rather than tumor (Section 8.2). Circulating miRNAs may therefore reflect the functional state of the tumor and immune compartment, but this remains a hypothesis rather than an established fact, and demonstrating it requires studies with paired tumor and plasma measurement, which do not currently exist. Establishing tissue-to-plasma concordance for the candidate miRNAs is a precondition for, not a detail of, their clinical development.

### 5.3 The neoadjuvant chemoimmunotherapy setting: the critical gap

Neoadjuvant chemoimmunotherapy has become standard care for resectable NSCLC. Across the landmark trials (CheckMate-816, KEYNOTE-671, CheckMate-77T, and AEGEAN), pCR rates range from roughly 17% to 25% and MPR rates from 30% to 37% compared with chemotherapy alone [6,7,9,10]. Both endpoints are strongly associated with improved event-free survival irrespective of subsequent adjuvant immunotherapy [12]. These numbers carry a clinical corollary that is easy to lose sight of: 70% to 80% of patients do not achieve pCR. All of them receive the same regimen, with its toxicity, surgical delay, and cost. Who are those 70% to 80%? Current tools cannot reliably identify them in advance.

PD-L1 TPS has modest predictive value for pCR but fails at the individual patient level. TMB has been inconsistent across neoadjuvant trials. ctDNA clearance during treatment is promising; in the NADIM program, undetectable ctDNA after neoadjuvant chemoimmunotherapy outperformed radiologic response for survival prediction [92], and ctDNA has been shown to track molecular patterns of disease progression in other NSCLC settings [93]. The limitation is that ctDNA measures residual tumor burden and genotype, not immune function. PET metabolic response correlates with pCR but remains an indirect readout. TCR diversity and TIL composition are still exploratory [15,94].

Until very recently, circulating miRNAs were absent from this list. A single study, published in late 2025 by Zhai et al., has now begun to address this gap. Using plasma exosomal miRNA profiling in 40 patients from a prospective neoadjuvant immunotherapy trial (NCT05244837), the authors identified a five-miRNA signature (let-7e-5p, miR-181a-2-3p, miR-1271-5p, miR-22-3p, miR-589-5p) that discriminated pCR from non-pCR with an AUC of 0.826 [95]. This is a proof of concept, but the small sample size, single-center design, and absence of an independent validation cohort mean the gap remains largely open: no adequately powered, multi-center study with external validation has yet been reported.

The neoadjuvant setting is well suited to biomarker development. Pathologic response provides a definitive, tissue-based endpoint, more objective than radiologic assessment. Serial blood draws at baseline, mid-treatment, and before surgery are already part of routine practice, which makes dynamic monitoring feasible without additional burden.

## 6 Circulating miRNAs versus ctDNA: comparative strengths, limitations, and complementarity

### 6.1 ctDNA: current status and strengths in NSCLC

Circulating tumor DNA is, at present, the most clinically advanced liquid biopsy analyte in NSCLC. Several FDA-approved ctDNA-based companion diagnostic assays are available for mutation detection, including the Cobas EGFR Mutation Test v2, Guardant360 CDx, and FoundationOne Liquid CDx, with the last providing comprehensive genomic profiling across 324 cancer genes and validated analytical performance (positive percent agreement 96.3%, negative percent agreement >99.9%) [96].

The clinical utility of ctDNA in NSCLC spans several domains. It allows non-invasive detection of actionable driver mutations (EGFR, ALK, ROS1, KRAS G12C) when tissue is insufficient or unavailable, and it identifies acquired resistance mechanisms (EGFR T790M and C797S, among others) that guide sequential therapy decisions. For the neoadjuvant context, the most relevant application is minimal residual disease (MRD) detection and treatment response monitoring. The short plasma half-life of ctDNA (approximately 1 to 2 h) makes it well placed to capture near-real-time fluctuations in tumor burden [97]. In the NADIM trial, ctDNA clearance after neoadjuvant chemoimmunotherapy outperformed radiologic response in predicting overall survival, which set the precedent for ctDNA-based dynamic monitoring in the perioperative immunotherapy setting [92]. The recently published final 5-year overall survival analysis of CheckMate-816 has now confirmed this with mature data: pre-surgical ctDNA clearance was associated with 5-year overall survival of 75.0% compared with 52.6% among patients without clearance, and the benefit was observed regardless of treatment arm [6].

### 6.2 Limitations of ctDNA that circulating miRNAs May address

The main limitations are low sensitivity in early-stage and low-tumor-burden disease. Until recently, ctDNA detection rates in stage I NSCLC were estimated at only 40% to 50% with standard assays, leaving a substantial proportion of patients with resectable disease uninformative at baseline. Even with the ultrasensitive NeXT Personal platform (whole-genome-based, tumor-informed, LOD95 of 1 to 3 ppm), preoperative ctDNA was detected in only 53% of patients with pathological stage I lung adenocarcinoma in the TRACERx cohort, and remained undetectable in nearly half [98]. The ceiling is inherent to ctDNA biology: detection depends on tumor cell death and DNA shedding, which are minimal in small, indolent tumors [99]. Circulating miRNAs are detectable in essentially all patients regardless of stage, because the actively secreted vesicular fraction of the circulating pool is not contingent on tumor cell death (Section 2.2, Table 4) [26].

**Table 4:** Circulating miRNAs versus ctDNA as liquid biopsy analytes in NSCLC: head-to-head comparison.

Feature	ctDNA	Circulating miRNAs
Biological information	Tumor genotype: driver mutations, resistance mechanisms, mutational burden	Functional phenotype: immune activation, EMT status, drug resistance pathways, PD-L1 regulatory state
Cellular origin	Tumor cells exclusively (via apoptosis/necrosis)	Multi-cellular: tumor, immune cells, platelets, stromal cells
Release mechanism	Passive (tumor cell death → DNA shedding); half-life ∼1-2 h	Both passive (AGO2 complexes, ∼90%) and active (exosomal secretion, ∼10%)
Detection in stage I NSCLC	∼53% with ultrasensitive assays (NeXT Personal, LOD95 1-3 ppm; Black et al., Nat Med 2025 [98])	Detectable in virtually all patients regardless of stage [100]
Confounders	CHIP: 42.3% of patients harbor ≥1 CH variant among reportable genes (Magee et al., CCR 2025 [100])	Hemolysis (affects up to 50% of measured miRNAs), age, sex, smoking, comorbidities, medications [42,101]
Capture of immune response	No, reflects tumor genotype, not tumor-immune interactions [13]	Potentially: miRNAs from immune and stromal cells may reflect host response; miR-34a, miR-200/141, and miR-197 regulate PD-L1 in tissue models, though tissue-to-plasma concordance is unproven [87,89,91]
Actionable mutation detection	Yes: EGFR, ALK, ROS1, KRAS G12C, resistance mechanisms (T790M, C797S)	No, cannot identify driver mutations or specific resistance mechanisms
MRD detection	Established: ctDNA clearance predicts OS in neoadjuvant setting (CheckMate-816 [6], NADIM [92])	Not validated for MRD; post-surgical miRNA decline demonstrated [102] but not tested head-to-head
Dynamic monitoring	Suitable, short half-life enables near-real-time burden tracking	Suitable, serial profiles correlate with RECIST response [41]; on-treatment miR-21 rises signal emerging resistance [103]
Regulatory status	3 FDA-approved companion diagnostics in NSCLC (Cobas EGFR v2, Guardant360 CDx, FoundationOne Liquid CDx)	No FDA- or EMA-approved assay for any cancer indication
Standardization maturity	More advanced: standardized variant calling, allele frequency reporting; BLOODPAC consortium active [104]	Less advanced: no consensus normalization, no certified reference materials, inter-laboratory ring trials advocated but not implemented [34,45]
Cost per assay	High: comprehensive genomic profiling requires NGS (€1500-3000+)	Low: qRT-PCR panels (∼€50-200); NGS-based profiling costs more but remains cheaper than ctDNA CGP
Platform requirements	NGS with sophisticated bioinformatics pipelines; centralized laboratory infrastructure	qRT-PCR widely available; ddPCR and nCounter emerging; decentralized testing feasible
Complementary value in neoadjuvant setting	ctDNA clearance → residual tumor burden (quantitative)	miRNA kinetics → quality of anti-tumor immune response (qualitative/functional)

Clonal hematopoiesis of indeterminate potential (CHIP) compounds the problem. Age-related somatic mutations in hematopoietic progenitor cells, particularly in TP53, DNMT3A, and TET2, can produce cfDNA variants indistinguishable from tumor-derived ctDNA in the absence of paired white blood cell sequencing. A recent analysis of over 16,000 liquid biopsy profiles found that 42.3% of patients harbored at least one CHIP variant among reportable clinical genes [105]. In the lung cancer population, where median age at diagnosis exceeds 65 years and smoking exposure is common, CHIP prevalence is high, complicating MRD interpretation and potentially generating false-positive results. The BLOODPAC Consortium established a dedicated CH/CHIP Working Group in 2024 specifically to address the standardization gap this confounder creates [104].

A deeper limitation is that ctDNA reflects tumor genotype and residual burden but does not capture the functional state of the tumor and immune microenvironment. It cannot directly inform on immune activation status, PD-L1 regulation, EMT dynamics, or treatment-induced immunomodulation, the biological processes that drive response to immunotherapy and chemoimmunotherapy. The inconsistent performance of ctDNA-derived TMB in predicting immunotherapy outcomes is a symptom of the same problem: genotypic information alone does not capture the complexity of tumor and immune interactions [13].

Cost and technical complexity of NGS-based ctDNA assays add a practical layer. Comprehensive genomic profiling assays are expensive and require sophisticated bioinformatics infrastructure, which limits scalability in resource-constrained healthcare systems [106].

### 6.3 Where circulating miRNAs offer distinct advantages

The advantages of circulating miRNAs over ctDNA are most visible in the places where ctDNA falls short. ctDNA reveals the tumor's mutational landscape, its genotype; circulating miRNAs reflect the regulatory state of both tumor and host cells, including immune activation, EMT status, and drug resistance pathway engagement [17,107]. The split between genotypic and functional information is the conceptual hinge on which the two analyte classes diverge in predictive value.

Three features underpin that complementary value. miRNA release is not contingent on tumor cell death: active exosomal secretion means that early-stage tumors undetectable by ctDNA can still generate informative miRNA profiles [26]. A substantial fraction of circulating miRNAs originates not from the tumor itself but from immune and stromal cells responding to it, and for immunotherapy prediction that multi-cellular origin is more useful than limiting [79]. The distinction that matters is between signal and contamination: miRNAs released by immune and stromal cells engaged with the tumor report on the host response, whereas those arising from erythrocyte and platelet lysis during sample handling do not, which is why the hemolysis controls set out in Section 3.1 are a precondition for reading this fraction as biology (Section 8.2). And qRT-PCR-based miRNA quantification is an order of magnitude cheaper than NGS-based ctDNA analysis, a practical point with direct consequences for global accessibility [108].

### 6.4 Limitations of circulating miRNAs relative to ctDNA

Many circulating miRNAs (miR-21 is the most familiar example) are elevated across multiple cancer types and non-malignant conditions including inflammation, cardiovascular disease, and metabolic disorders, which limits their specificity for NSCLC as standalone biomarkers. ctDNA carries tumor-specific mutations that provide unambiguous identification of the cancer genotype [49,109].

No FDA- or EMA-approved circulating miRNA assay exists for any cancer indication. ctDNA has established regulatory precedent through multiple approved companion diagnostics, while circulating miRNAs have not yet moved through the regulatory pathway. The disparity reflects biological challenges, but it also reflects the field's failure to achieve adequate standardization [34]. The unresolved normalization and pre-analytical issues discussed earlier continue to impede cross-study comparability and clinical translation. ctDNA quantification has its own pre-analytical difficulties, but standardization of variant calling and allele frequency reporting is further along than the equivalent work for miRNA expression [30].

Circulating miRNAs also cannot identify actionable driver mutations or specific resistance mechanisms. A patient with an EGFR-activating mutation still requires ctDNA or tissue-based genotyping for targeted therapy selection; miRNAs provide functional and regulatory information that complements, but does not replace, genotypic data [110].

### 6.5 The complementarity argument: toward multimodal liquid biopsy

Read together, the evidence supports a framework in which ctDNA and circulating miRNAs are complementary rather than competing analytes: ctDNA provides genotypic data and MRD detection, miRNAs provide functional immune phenotyping and treatment-response prediction [110]. The complementarity is most useful in the neoadjuvant chemoimmunotherapy setting, where ctDNA clearance captures residual tumor burden while miRNA kinetics may capture the quality of the immune response elicited by treatment (Table 4). These are separately informative dimensions of pathologic response. Preliminary support for such multimodal approaches has been documented [106], but prospective validation has not yet been done.

## 7 Dynamic monitoring: circulating miRNAs as real-time surrogates of treatment response

Most circulating miRNA biomarker research in NSCLC has relied on single-timepoint, cross-sectional designs: a baseline blood draw compared against healthy controls or correlated with a fixed clinical outcome. This approach has yielded the prognostic and predictive associations that the literature reports, but it sets aside what may be the most clinically valuable property of circulating biomarkers, namely the capacity for serial, longitudinal measurement that tracks the kinetics of treatment response over time.

### 7.1 Circulating miRNA dynamics during systemic therapy

The first systematic evidence that circulating miRNA profiles shift in concordance with treatment response during systemic therapy in NSCLC came from Abdipourbozorgbaghi et al., who performed serial plasma miRNA quantification with paired blood draws obtained before and during systemic therapy. Using radiologic assessment per RECIST 1.1 as the reference standard (mean interval of 13 days between blood draw and imaging), the authors found a directional concordance: NSCLC-associated circulating miRNAs decreased in patients with radiologic tumor response and increased in those with disease progression [41]. The pattern held across both LUAD and LUSC subtypes and across the differentially expressed miRNA panels specific to each histotype. One discordant case is worth noting: a stage IIIA patient receiving neoadjuvant chemotherapy in whom differentially expressed miRNAs paradoxically increased despite radiologic response [41]. The observation is anecdotal, but it raises a real question about whether the temporal dynamics of miRNA release differ between the neoadjuvant and palliative treatment settings.

The EGFR-TKI context is different from chemoimmunotherapy, but the study by Leonetti et al. is worth pulling in here because it is one of the few in NSCLC to use truly serial measurement with paired samples at defined timepoints. In 39 EGFR-mutated advanced NSCLC patients, plasma miR-21, miR-27a, and miR-181a were measured at baseline and after two months of EGFR-TKI therapy. Patients with stable disease showed a significant on-treatment increase in circulating miR-21 (fold change 2.6, P = 0.029) compared with those achieving partial or complete response; miR-27a and miR-181a followed a similar but non-significant trend (fold changes 3.1 and 2.0, respectively) [103]. The accompanying preclinical data showed that miR-21 levels rose in NSCLC cell lines acquiring EGFR-TKI resistance, which gives a mechanistic basis for the clinical observation. The pattern is consistent with rising circulating miR-21 during treatment acting as an early molecular signal of emerging resistance, potentially preceding radiologic progression [103].

### 7.2 Post-surgical miRNA decline and recurrence surveillance

A separate but convergent line of evidence shows that circulating miRNA levels decline after surgical tumor resection, a prerequisite for any biomarker intended to track treatment response. Asakura et al. developed a two-miRNA diagnostic model (miR-1268b and miR-6075) achieving 99% sensitivity and 99% specificity for resectable lung cancer in a large independent validation cohort (n = 1358 patients; n = 1970 non-cancer controls), though the case-control design, comparing diagnosed patients with non-cancer participants, would be expected to yield higher apparent performance than a screening population. The magnitude of the post-surgical change is substantial: the mean diagnostic index dropped from +0.98 ± 0.82 preoperatively to −4.30 ± 1.15 postoperatively (P < 0.001) in 180 patients, with each component miRNA showing individually significant decreases [102].

Leidinger et al. extended the longitudinal dimension, profiling 1205 miRNAs in plasma from 26 NSCLC patients at eight timepoints over 18 months following surgical resection, compared with 12 non-cancer controls. Patients who subsequently developed metastases had a higher number of detectable circulating miRNAs (P = 0.0096), while disease-free patients showed more pronounced expression-level changes relative to controls. Despite the modest cohort, this remains the only NSCLC study to have tracked circulating miRNAs longitudinally over 18 months post-resection, a gap that is hard to explain given that the technical feasibility was demonstrated over a decade ago [111]. That detectable miRNA counts differ between patients who recur and those who do not is reason enough to ask whether post-surgical miRNA profiling could sit alongside ctDNA-based minimal residual disease surveillance, though no direct comparison has been done.

### 7.3 Implications for the neoadjuvant chemoimmunotherapy setting

The rationale for studying circulating miRNAs in the neoadjuvant setting has been laid out in Section 5.3. What dynamic monitoring adds, specifically, is the capacity to capture treatment response trajectories rather than static baseline snapshots. If miRNA kinetics during chemoimmunotherapy correlate with pathologic response, as the directional concordance with RECIST observed by Abdipourbozorgbaghi et al. suggests they might [41], serial measurement could flag non-response earlier than imaging and open a window for adaptive treatment decisions (Fig. 2 illustrates possible points where the treatment plan could be reassessed and adapted individually).

**Fig. 2:**
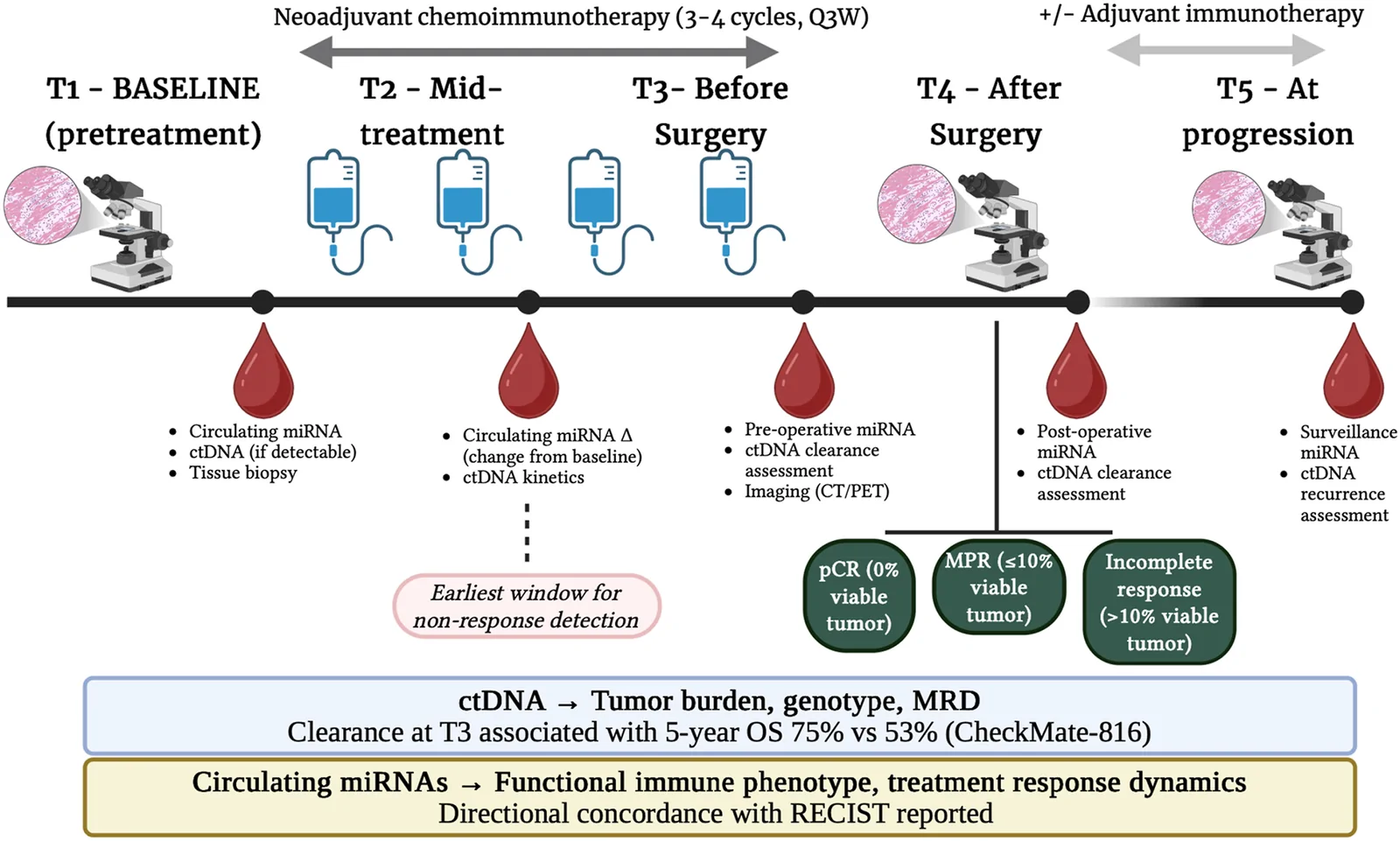
**Conceptual framework for dynamic circulating biomarker monitoring during neoadjuvant chemoimmunotherapy in resectable NSCLC.** Five clinically practical sampling timepoints are shown: baseline (T1, pre-treatment), mid-treatment (T2, cycle 2 to 3), before surgery (T3), after surgery (T4), and at progression (T5). At baseline, circulating miRNA profiling, ctDNA assessment, and tissue-based biomarker evaluation (PD-L1, TMB) establish the pre-treatment landscape. Mid-treatment sampling represents the earliest window for detecting non-response through changes in circulating miRNA profiles (Δ from baseline) and ctDNA kinetics. Pre-operative sampling captures the integrated treatment effect, including ctDNA clearance status, which has been associated with 5-year overall survival of 75.0% versus 52.6% in CheckMate-816 [6]. Pathologic response at surgical resection (pCR, MPR, or no MPR) provides the tissue-based endpoint, after which post-surgical sampling supports minimal residual disease assessment and sampling at progression documents the molecular profile of relapse. The lower panel illustrates the complementary biological information captured by each analyte class: ctDNA reflects tumor genotype, mutational burden, and minimal residual disease, while circulating miRNAs may reflect the functional state of tumor and immune interactions and the dynamics of treatment response [13,17]. Integration of ctDNA clearance with miRNA kinetics into multimodal models could provide more predictive power than either analyte alone. Created in BioRender.

## 8 Challenges, limitations, and barriers to clinical translation

### 8.1 The reproducibility crisis in circulating miRNA biomarker research

Nearly two decades have passed since Mitchell et al. showed that circulating miRNAs are stable and detectable in plasma [19]. In that time, the field has not converged on a reproducible set of validated circulating miRNA biomarkers for any cancer type, NSCLC included. This is not a small problem. Irreproducibility is what the two failures described earlier produce together: the pre-analytical variability that is the largest single source of measurement noise (Section 3.1), and the absence of a consensus normalization reference that leaves that noise uncorrected (Section 3.3). It is what stands between a promising biomarker class and clinical use.

The evidence for the claim is direct. Independent groups studying the same disease in the same specimen type routinely identify entirely non-overlapping candidate miRNAs [33,34]. Lakkisto et al. traced that discordance primarily to technical variability introduced at every stage of the analytical workflow, from specimen handling to data analysis, rather than to biology alone [34]. Greytak et al. reached the same conclusion: the absence of standardized specimen handling is a central driver of irreproducible results [33].

When pre-analytical variation is compounded by differences in analytical platforms and normalization strategies, cumulative technical noise can overwhelm the biological signal [30,32]. The miRQC study showed this explicitly: cross-platform concordance is worst for low-abundance miRNAs, which is the concentration range of most putative circulating biomarkers [35]. More recently, Sopić et al. have advocated inter-laboratory ring trials to address this, noting that certified reference materials for miRNA quantification do not yet exist, a gap that on its own could delay clinical adoption substantially [45].

### 8.2 Biological confounders

Technical variability is only half the problem. Circulating miRNA profiles are shaped by non-malignant variables that most NSCLC biomarker studies fail to adequately control: age, sex, smoking status, BMI, comorbidities, concomitant medications, circadian rhythm, and physical activity all exert measurable effects [30,32]. Without rigorous matching or statistical adjustment, these confounders introduce bias that can inflate or mask genuine tumor-derived signals.

Hemolysis deserves its own scrutiny. Pritchard et al. examined 79 solid tumor circulating miRNA biomarkers reported in the literature and found that 58% were highly expressed in blood cells. Blood cell count perturbations and hemolysis altered plasma miRNA levels by up to 50-fold, leading the authors to warn that many reported cancer biomarkers may reflect hematologic phenomena rather than tumor biology [42]. Kirschner et al. extended that work, showing that even low-grade hemolysis, below the threshold of visual detection, materially alters the concentration of numerous miRNAs previously proposed as disease biomarkers, including miR-21, miR-106a, miR-92a, and miR-17 [101].

### 8.3 Statistical and study design limitations

Sample size is the most visible problem. Multi-miRNA panel discovery involves high-dimensional data, hundreds or thousands of candidate miRNAs, measured in cohorts of fewer than 100 patients [34]. The events-per-variable ratio, which underpins model stability, is routinely violated [34]. When machine learning algorithms are layered onto these small datasets, the risk of overfitting rises sharply [112]. Many studies report promising AUC values from internal cross-validation but never confirm them in independent, geographically and temporally distinct cohorts. The TRIPOD + AI guidelines, published in 2024, set updated standards for transparent reporting of prediction models developed using both regression and machine learning methods [44]. These standards are rarely followed in the circulating miRNA literature.

Publication bias compounds the problem. Null findings and failed replications are less likely to reach print, and selective reporting of only statistically significant miRNAs from larger panels, without correction for multiple testing, inflates apparent biomarker performance [34].

The most consequential gap is conceptual. A statistically significant association (p < 0.05, hazard ratios of 1.5 to 2.5) does not translate by default into clinical usefulness at the individual patient level. A biomarker that is associated with an outcome is not the same thing as a biomarker that provides information one can act on at the bedside [34,41].

## 9 Future directions

### 9.1 Methodological innovation: from static to dynamic biomarkers

Continuing to assess individual circulating miRNAs at a single timepoint, and expecting clinically meaningful results to follow, is no longer a defensible strategy. Progress requires a shift toward designs built around three interrelated priorities.

Multi-miRNA panel signatures with demonstrated complementarity are the most immediate need. Individual circulating miRNAs, even those with sound biological rationale, are unlikely to achieve the sensitivity and specificity that clinical decision-making demands. The redundancy built into miRNA regulatory networks, where a single miRNA may target hundreds of transcripts and a single transcript may be regulated by dozens of miRNAs, makes a combinatorial approach essential [113]. The histology-specific panels described in Section 4.3 illustrate multi-analyte signatures of that kind, and the miRisk score provides proof of concept that multi-cohort validation of miRNA signatures is achievable [13,41]. These studies sketch the trajectory toward clinically actionable multi-analyte signatures. What separates a defensible panel from a statistically convenient one is biological plausibility: future panels should be built from miRNAs with mechanistically established roles in the pathways targeted by the intervention under investigation, rather than assembled by data-driven selection alone. A prerequisite that sits upstream of panel design is establishing, for each candidate miRNA, whether its circulating level tracks its intratumoural level; paired tumor and plasma measurement is almost entirely absent from the current literature and should be built into discovery-phase studies. The neoadjuvant window is unusually well suited to this, since a resection specimen is obtained by design, allowing paired tissue and plasma sampling at a defined timepoint without additional procedures.

The second priority is longitudinal measurement that tracks treatment response kinetics rather than offering a single static snapshot. Preliminary evidence shows that changes in circulating miRNA profiles correlate with radiologic tumor response per RECIST 1.1, and that post-surgical decreases in NSCLC-associated miRNAs may signal successful resection, with subsequent rises potentially marking early recurrence [41]. In the neoadjuvant chemoimmunotherapy setting, where the case for miRNA-based prediction has been laid out in Section 5.3, dynamic measurement at defined timepoints could allow identification of non-responders early enough for therapeutic strategy to be reconsidered.

The third priority is integration with complementary liquid biopsy analytes: ctDNA clearance, exosomal PD-L1, and T-cell receptor (TCR) repertoire profiling [18,94]. As argued in Section 6, ctDNA and miRNAs capture orthogonal dimensions of treatment response, so a multimodal model combining both is likely to provide more predictive power than either analyte alone.

### 9.2 Multi-omics integration and computational approaches

Treatment response in NSCLC is driven by the interplay of tumor genetics, epigenetics, the immune microenvironment, and host physiology, a complexity that no single analyte class is likely to capture on its own. Integrating miRNA profiles with genomic, transcriptomic, proteomic, and metabolomic data layers is a logical extension of the panel-based approaches described above. Liu et al. showed that adding a three-exosomal-miRNA signature (miR-23b-3p, miR-10b-5p, miR-21-5p) to clinical prognostic variables improved the 12-month time-dependent AUC for survival prediction from 0.88 to 0.91 (P = 0.015) in 196 NSCLC patients, a modest but statistically significant increment that illustrates the additive value of molecular data on top of clinical variables [114].

Machine learning offers tools for identifying non-linear miRNA signature patterns that traditional statistical methods may miss, but the promise comes with risks that are by now well known. Overfitting is a particular concern given the small sample sizes typical of miRNA biomarker studies. The updated TRIPOD + AI guidelines provide the reporting framework needed to ensure that computationally derived signatures are generalizable in any real sense: rigorous cross-validation, temporally and geographically independent test sets, and transparent disclosure of model performance [44]. Without that discipline, even sophisticated algorithms risk producing artifacts of data-driven discovery rather than clinically meaningful predictors. The recent Jurj et al. perspective has made the point directly, noting that AI-driven analyses can advance miRNA biomarker development, but only when accompanied by sufficient training data and methodological rigor to prevent inflated performance estimates [113].

One caveat is that no amount of sophisticated downstream analysis can compensate for variability introduced at the pre-analytical stage. The point is widely acknowledged in the field, but it remains poorly addressed in practice.

### 9.3 Standardization as a prerequisite for progress

None of the directions above can succeed without addressing the underlying problem of methodological standardization. The work that is needed is multi-center, prospective, and built on the pre-analytical and analytical standards described earlier [30,33]. Inter-laboratory ring trials, as Sopić et al. have advocated, should become a routine part of multi-center biomarker study design, giving quantitative estimates of inter-institutional variability and a basis for reducing it [45]. Without that commitment, even carefully designed studies risk producing institution-specific findings that cannot be replicated elsewhere.

## 10 Conclusions

Circulating miRNAs have shown prognostic associations in NSCLC and a developing predictive value for both chemotherapy and immunotherapy outcomes. Their biological strengths, stability in biofluids, the potential to phenotype tumor and immune interactions functionally, and access to host response dimensions that genotype-based assays cannot reach, are reason enough to keep investigating them. Clinical translation, however, is still held back by methodological heterogeneity and the near-complete absence of large-scale prospective validation.

The neoadjuvant chemoimmunotherapy setting is an underexplored and clinically important context in which circulating miRNAs could address a real unmet need for non-invasive predictive biomarkers of pathologic response. Closing the distance between the current fragmented evidence and clinical utility will require demonstration that circulating levels track their intratumoural counterparts, biologically informed panel design, longitudinal measurement, multimodal liquid biopsy integration, and methodological rigor that the field has not yet imposed on itself. More exploratory, retrospective, single-center studies will not get the field there. Hypothesis-driven, adequately powered, prospective investigations, designed to meet the evidentiary standards that clinical implementation actually requires.

## Statement for studies in humans/animals

This article is a narrative review of previously published literature. It does not report any studies involving human participants, human data, human tissue, or animals performed by any of the authors. Accordingly, ethics committee approval, patient consent to participate, and consent for publication were not required. All primary studies cited in this review were conducted by their respective authors in accordance with the ethical standards of their institutions and with the 1964 Declaration of Helsinki and its later amendments, as reported in the original publications.

## Statement for studies in humans/animals

This article is a narrative review of previously published literature. It does not report any studies involving human participants, human data, human tissue, or animals performed by any of the authors.

Accordingly, ethics committee approval, patient consent to participate, and consent for publication were not required. All primary studies cited in this review were conducted by their respective authors in accordance with the ethical standards of their institutions and with the 1964 Declaration of Helsinki and its later amendments, as reported in the original publications.

## Ethics approval and consent to participate

Not applicable. This article is a narrative review of previously published literature and does not report any study involving human participants, human data, human tissue, or animals.

## Consent for publication

Not applicable.

## Availability of data and materials

Data sharing is not applicable to this article as no datasets were generated or analysed during the current study.

## Funding

This research received no specific grant from any funding agency in the public, commercial, or not-for-profit sectors.

## Declaration of competing interests

Iolanda Augustin, Adelina Birceanu, and Laura Mazilu report personal fees (honoraria for lectures, presentations, speaker bureaus, or advisory boards) from various pharmaceutical companies and have participated as investigators in industry-sponsored clinical trials, all outside the submitted work. Veronica Manolache reports personal fees (honoraria for lectures, presentations, or advisory boards) from various pharmaceutical companies, outside the submitted work. Ileana Constantinescu declares no competing interests.

No author received funding or support related to the present study, which was conducted without industry involvement.

## List of abbreviations

3′-UTR: 3′ untranslated region
AGO2: Argonaute 2
ALK: anaplastic lymphoma kinase
AUC: area under the curve
BLOODPAC: Blood Profiling Atlas in Cancer
BMI: body mass index
CDx: companion diagnostic
cfDNA: cell-free DNA
CGP: comprehensive genomic profiling
CH: clonal hematopoiesis
CHIP: clonal hematopoiesis of indeterminate potential
CI: confidence interval
CT: computed tomography
ctDNA: circulating tumor DNA
ddPCR: droplet digital PCR
DFS: disease-free survival
DGCR8: DiGeorge syndrome critical region 8
EFS: event-free survival
EGFR: epidermal growth factor receptor
EMA: European Medicines Agency
EMT: epithelial-mesenchymal transition
EPV: events per variable
ESCRT: endosomal sorting complex required for transport
EV: extracellular vesicle
FDA: US Food and Drug Administration
HDL: high-density lipoprotein
HIF-1α: hypoxia-inducible factor 1-alpha
hnRNP A2/B1: heterogeneous nuclear ribonucleoprotein A2/B1
HR: hazard ratio
ICI: immune checkpoint inhibitor
LOD95: 95% limit of detection
LUAD: lung adenocarcinoma
LUSC: lung squamous cell carcinoma
miRNA: microRNA
miRQC: microRNA quality control
MPR: major pathologic response
mRNA: messenger RNA
MRD: minimal residual disease
NGS: next-generation sequencing
NSCLC: non-small cell lung cancer
OR: odds ratio
OS: overall survival
pCR: pathologic complete response
PD-1: programmed cell death protein 1
PD-L1: programmed death-ligand 1
PET: positron emission tomography
PFS: progression-free survival
pre-miRNA: precursor microRNA
pri-miRNA: primary microRNA
Q3W: every three weeks
qRT-PCR: quantitative reverse transcription polymerase chain reaction
RECIST: Response Evaluation Criteria in Solid Tumors
REMARK: Reporting recommendations for tumor marker prognostic studies
RISC: RNA-induced silencing complex
RNA-seq: RNA sequencing
RNase: ribonuclease
ROC: receiver operating characteristic
ROS1: ROS proto-oncogene 1
SD: standard deviation
TCGA: The Cancer Genome Atlas
TCR: T-cell receptor
TIL: tumor-infiltrating lymphocyte
TKI: tyrosine kinase inhibitor
TMB: tumor mutational burden
TPS: tumor proportion score
TRBP: transactivation-responsive RNA-binding protein
TRIPOD + AI: Transparent Reporting of a multivariable prediction model for Individual Prognosis Or Diagnosis, extended for Artificial Intelligence
